# Lipopeptide Biosurfactant Pseudofactin II Induced Apoptosis of Melanoma A 375 Cells by Specific Interaction with the Plasma Membrane

**DOI:** 10.1371/journal.pone.0057991

**Published:** 2013-03-06

**Authors:** Tomasz Janek, Anna Krasowska, Agata Radwańska, Marcin Łukaszewicz

**Affiliations:** 1 Department of Biotransformation, Faculty of Biotechnology, University of Wroclaw, Wroclaw, Poland; 2 Department of Cell Pathology, Faculty of Biotechnology, University of Wroclaw, Wroclaw, Poland; 3 Division of Chemistry and Technology Fuels, Wroclaw University of Technology, Wroclaw, Poland; University of Tennessee, United States of America

## Abstract

In the case of melanoma, advances in therapies are slow, which raises the need to evaluate new therapeutic strategies and natural products with potential cancer cell inhibiting effect. Pseudofactin II (PFII), a novel cyclic lipopeptide biosurfactant has been isolated from the Arctic strain of *Pseudomonas fluorescens* BD5. The aim of this study was to investigate the effect of PFII on A375 melanoma cells compared with the effect of PFII on Normal Human Dermis Fibroblast (NHDF) cells and elucidate the underlying mechanism of PFII cytotoxic activity. Melanoma A375 cells and NHDF cells were exposed to PFII or staurosporine and apoptotic death was assessed by monitoring caspase 3-like activity and DNA fragmentation. From time-dependent monitoring of lactate dehydrogenase (LDH) release, Ca^2+^ influx, and a correlation between Critical Micelle Concentration (CMC) we concluded that cell death is the consequence of plasma membrane permeabilisation by micelles. This finding suggests that pro-apoptotic mechanism of PFII is different from previously described cyclic lipopeptides. The mechanism of PFII specificity towards malignant cells remains to be discovered. The results of this study show that PFII could be a new promising anti-melanoma agent.

## Introduction

Melanoma is the most aggressive form of skin cancer with a median survival time of 8–9 months and a 3-year survival rate of 10%–15%. In Europe, dacarbazine (DTIC) is the only approved drug for use as a systemic therapies for melanoma lesions [1]–[4]. Most of the cytotoxic anticancer drugs in current use have been shown to induce apoptosis in susceptible cells [5]. Apoptosis, which is a major pathway of programmed cell death, plays essential roles in the maintenance of homeostasis and tissue development in organisms. The major apoptotic pathways have been identified as the death receptor-mediated pathway, the mitochondrial apoptotic pathway, and the endoplasmic reticulum pathway [6]–[9].

Biosurfactants are biological surface-active compounds with both hydrophilic and hydrophobic moieties produced by diverse microorganisms. Several bioactive natural surfactants, e.g., lipopeptide and glycolipid have been found to possess antibacterial, antifungal, anti-viral, hemolytic and ionophoric properties [10]–[12]. Some of these molecules have been shown to induce apoptosis in tumor cells. For example, a lipopeptide produced by *Bacillus subtilis* has anti-tumor activity on LoVo cells [13] and a cyclic lipopeptide from *Bacillus natto* T-2 induces apoptosis in human leukemia K562 cells [14] by an increase in [Ca^2+^] and Extracellular Signal-regulated Kinases (ERK) phosphorylation [15]. Glycolipids from *Candida antarctica* and their analogs have been implicated in growth arrest, apoptosis, and differentiation of mouse malignant melanoma and human HL60 cells [16], [17].

We have previously purified and identified two new cyclic lipopeptides – pseudofactin I and pseudofactin II from *Pseudomonas fluorescens* BD5 [18], [19]. Both compounds are cyclic lipopeptides (Gly-Ser-Thr-Leu-Leu-Ser-Leu-Leu/Val) with a palmitic acid connected to the terminal amino group of the octapeptide. The C-terminal carboxylic group of the last amino acid (Val or Leu) forms a lactone bond with the hydroxyl of Thr3. Previously we reported that pseudofactin II (PFII) lowered the adhesion and partially dislodged biofilm of five bacterial species and *Candida albicans* [20]. The aim of this study was to examine whether and how PFII affects melanoma cells. We have found that PFII induces apoptosis in melanoma (A375) cell line and that its biosurfactant activity is most effective above critical micelle concentration (130–140 µM).

## Results

### Cell Proliferation

**Figure 1:**
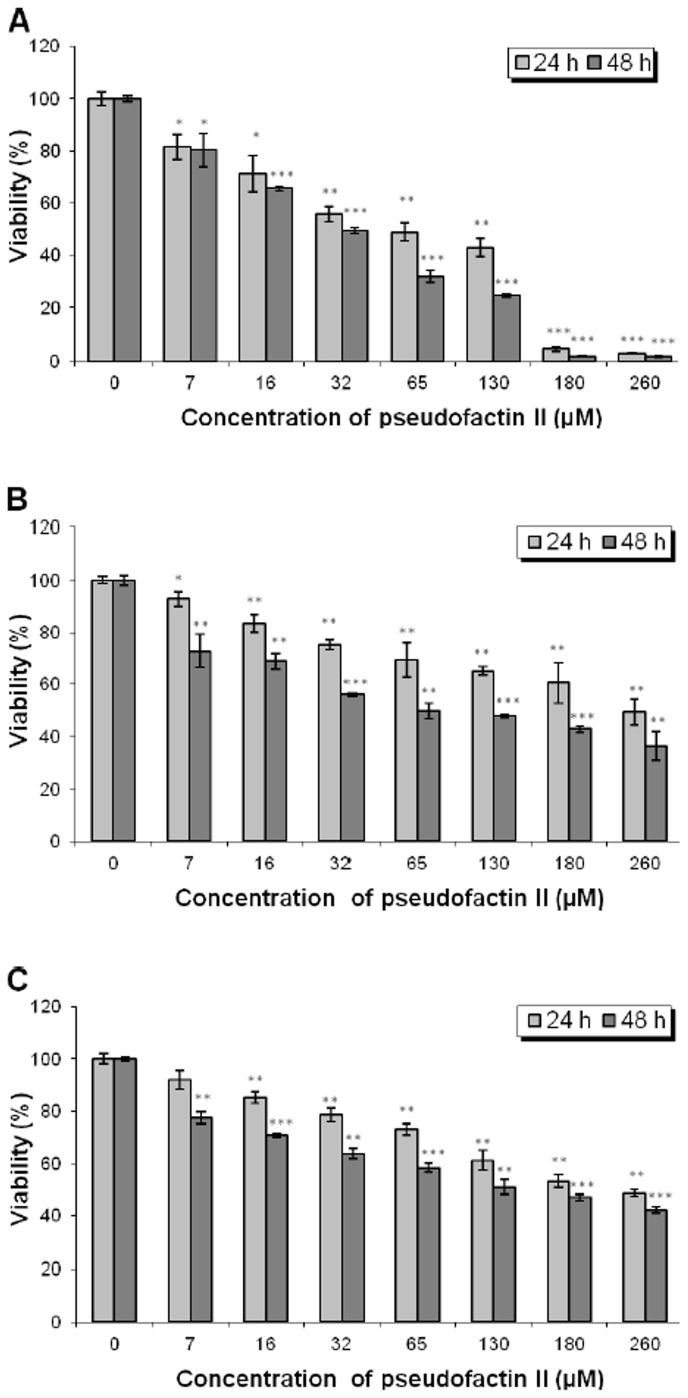
Proliferation rate of melanoma A375, NHDF and NHEK cells measured by MTT assay. A375 cells (A), NHDF cells (B) and NHEK cells (C) were treated with 7–260 µM concentrations of PFII for 24 h or 48 h, incubated with MTT and the number of viable cells measured spectrophotometrically at 570 nm. The bars represent the means ± SD of triplicate values for three independent experiments. *0.05>P>0.01, **0.01>P>0.001, ***P<0.001.

The proliferation rate of melanoma A375 cells and normal human dermal fibroblast (NHDF) cells grown in the presence of PFII was measured by MTT assay. A dose-dependent decrease in melanoma cell viability was observed with increasing (7 µM to 260 µM) concentrations of PFII (Figure 1A). The highest concentrations of PFII (180 µM and 260 µM) caused a nearly complete eradication of melanoma cells. In contrast, the viability and proliferation of NHDF and NHEK cells were less affected by PFII. NHDF and NHEK cells exposed to the highest PFII concentration (260 µM) and/or longer incubation times resulted in an approximate 50% decrease in proliferation rate (Figure 1B, C).

### Nuclei Fragmentation

**Figure 2:**
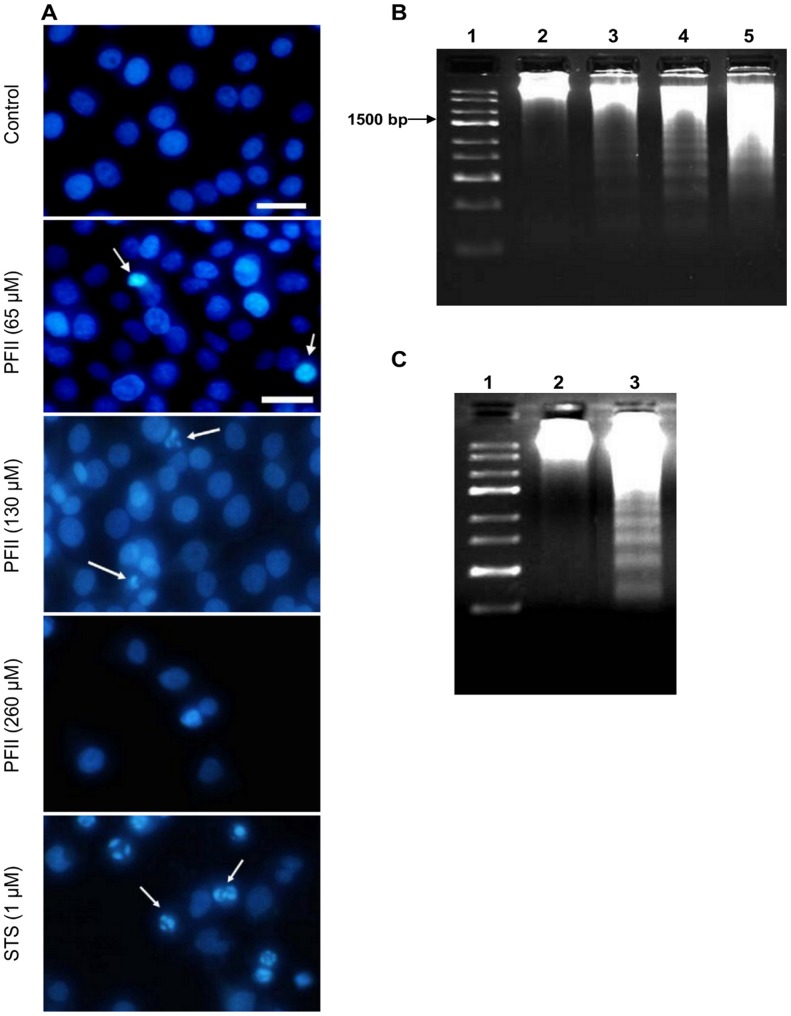
Nuclear morphology of apoptotic A375 cells. (A) The nuclei of PFA fixed PFII treated cells were stained with Hoechst 33342 and analyzed by fluorescence microscopy. Arrows indicate apoptotic nuclei. Scale bar: 20 µm. (B) Agarose gel electrophoresis of DNA fragmentation of melanoma A375 cells. Lane 1, DNA marker; Lane 2, No treatment; Lane 3, 4, 5, PFII at concentration 65, 130 and 260 µM for 24 h, respectively. (C) As a positive control for DNA laddering melanoma A375 cells were treated with 1 µM staurosporine (STS) for 2 h. Lane 1, DNA marker; Lane 2, No treatment; Lane 3, STS. Control cells were treated with an equivalent amount DMSO to a final concentration of 1%.

To evaluate the potential pro-apoptotic activity of PFII on melanoma A375 cells, the integrity of PFII-treated melanoma cell nuclei was examined using fluorescence microscopy after Hoechst 33342 staining (Figure 2A). The results were compared with those observed in the presence of staurosporine (STS), a known apoptotic agent. Melanoma cells grown in the presence of PFII or STS exhibited changes characteristic for apoptosis (Figure 2A, B, C), i.e., fragmented and ‘moon-shaped’ nuclei (Figure 2A, white arrows). We did not observe any changes in the nuclear integrity of NHDF cells cultured in the presence of the same concentrations of PFII (data not shown).

### Actin Condensation and Caspase-3 Activation

**Figure 3:**
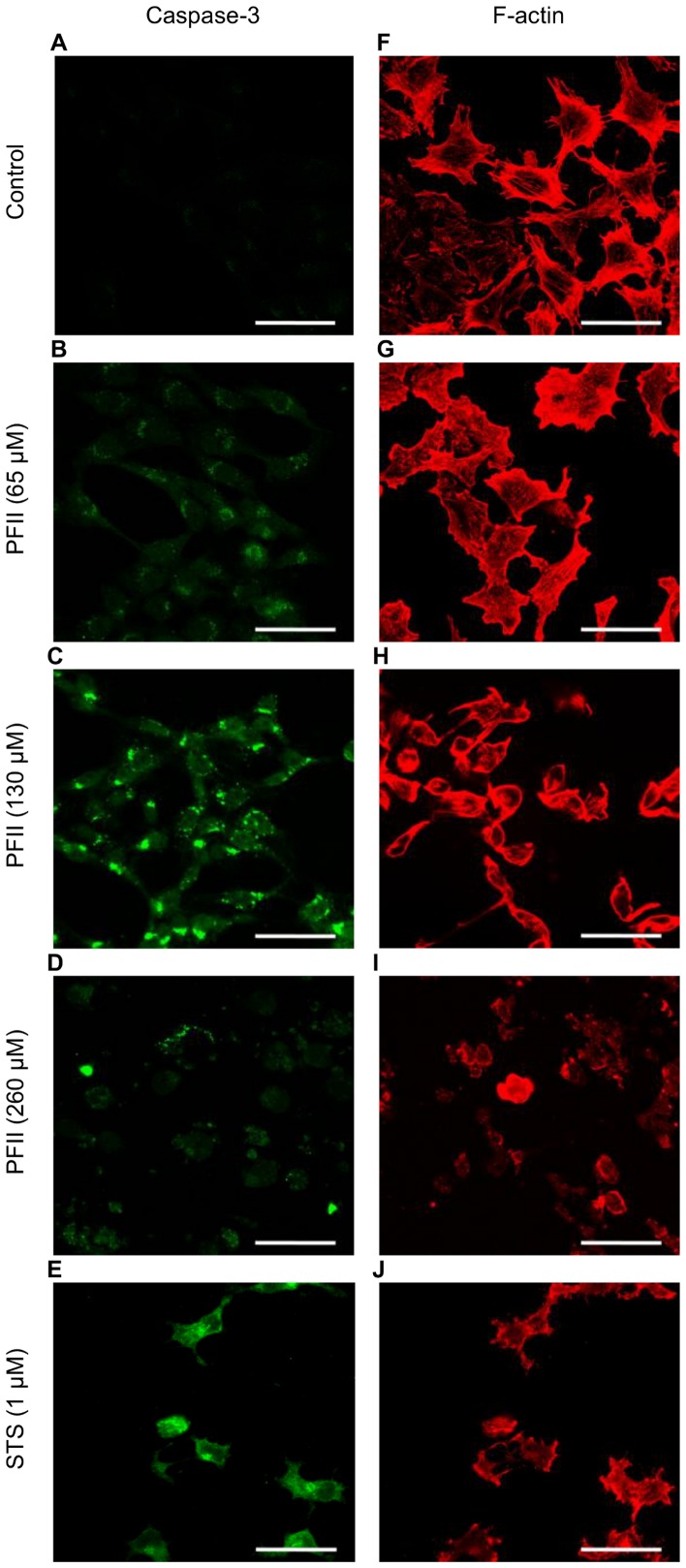
Caspase-3 activation and actin cytoskeletal organization in melanoma A375 cells cultured in the presence of PFII. Cells were grown on glass coverslips in the presence of 65, 130, 260 µM or 1 µM staurosporine (STS). (A–E) Active caspase-3 was visualized with anti-active caspase-3 antibody followed by a FITC-conjugated secondary antibody (green). (F–J) Actin was visualized using laser scanning confocal microscope (LSCM) after staining with Alexa Fluor 568 - conjugated phalloidin (red). Scale bar - 50 µm.

**Figure 4:**
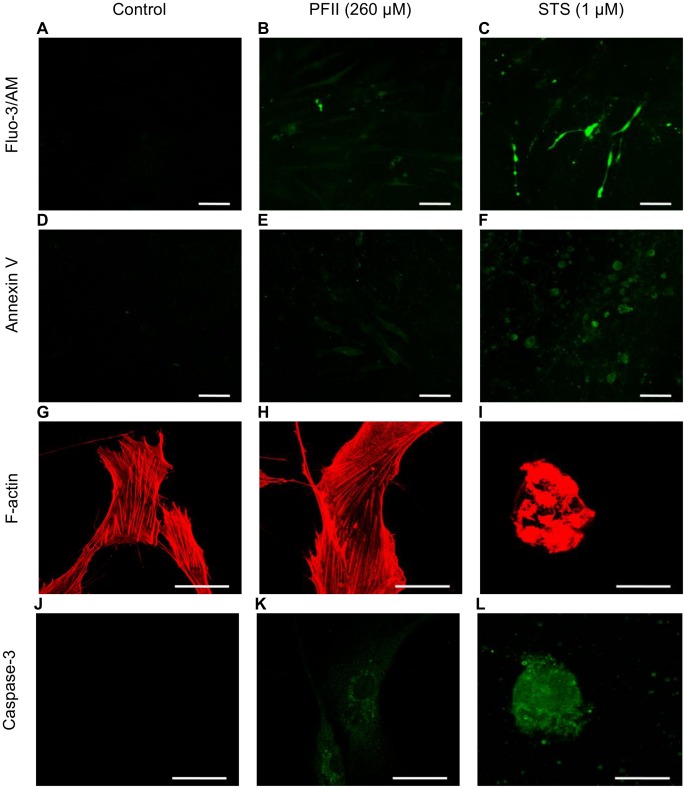
Apoptotic markers in PFII treated NHDFs. (A–C) [Ca^2+^]i fluorescence visualizations in cells as revealed by using an LSCM with fluorescent probe Fluo3/AM, scale bar - 100 µm. (D–F) Externalized phosphatidylserine by annexin V-fluorescein binding after PFII treatment, scale bar - 100 µm. Third (G–I) and fourth (J–L) panels show actin cytoskeletal organization and caspase-3 activation. Scale bar - 50 µm.

Melanoma A375 cells cultured in the presence of PFII reorganized their actin cytoskeleton. The lowest concentration of PFII (65 µM) employed induced a sub-membrane condensation of filamentous actin (F-actin). At higher concentrations of PFII (130–260 µM) the cells became rounded, exhibited F-actin disorganization, strong sub-membrane condensation, and bleb formation. The actin cytoskeleton of melanoma cells cultured in the presence of STS was almost completely disorganized and actin “aggregates” were visible, even at the lowest tested concentration (65 µM) after 24 hours of incubation (Figure 3F–J). The disruption of nuclear integrity, cyto-morphological changes, and actin cytoskeletal disorganization in melanoma cells grown in the presence of PFII were accompanied by caspase-3 activation similar to that observed in melanoma cells cultured in medium supplemented with STS (Figure 3A–E). The normal human dermal fibroblast, which were used as a control, did not respond to the PFII treatment with induction of any of the applied apoptotic markers. When comparing images G, H and J, K of Figure 4, we did not observe significant alterations in the distribution of F-actin and increase in caspase-3 activity in NHDF cells grown in medium with PFII. In contrast, staurosporine induced the apoptotic changes in NHDF cells (Figure 4 I, L).

### DNA Fragmentation

The effect of PFII on DNA fragmentation in melanoma cells is in Figure 2B. A “ladder” pattern representing fragmentation of melanoma cell DNA into oligonucleosome length fragments was observed after 24 h of PFII treatment. We also observed internucleosomal chromatin cleavage in melanoma cells treated with 1 µM staurosporine for 2 h (Figure 2C). Treatment of melanoma cells with PFII or staurosporine resulted in similar DNA fragmentation patterns.

### Calcium Influx

**Figure 5:**
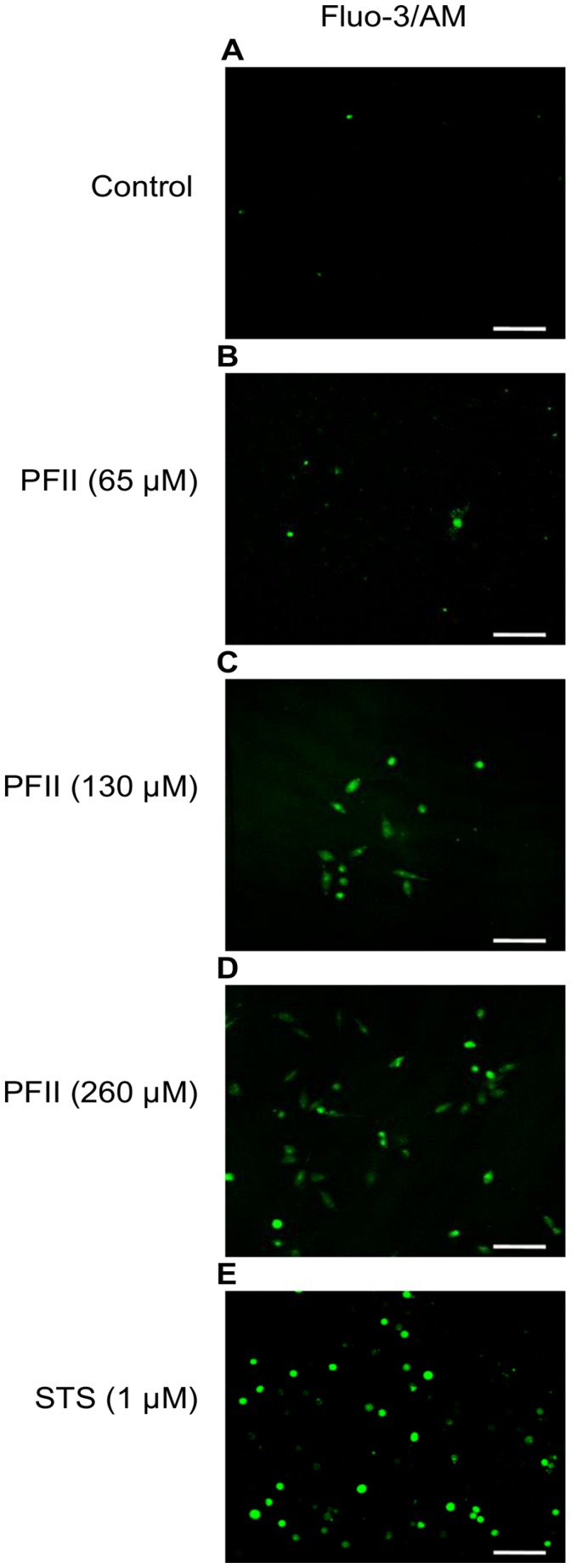
[Ca^2+^]*i* fluorescence visualizations and intensities in PFII-treated A375 cells. Intracellular calcium fluorescent signal in A375 cells as visualized by LSCM using the fluorescent probe Fluo-3/AM. (A) Control (untreated); (B–D) PFII for 24 h; (F) STS for 2 h. Scale bar - 100 µm.

Fluo-3-AM fluorescent probe labeled melanoma cells treated with PFII, as well as apoptotic cells in the presence of STS, exhibited significant Ca^2+^ influx (Figure 5). NHDF cells, which were used as a control, did not respond to PFII treatment with induction of the Fluo-3/AM (Figure 4A, B). In contrast, staurosporine was able to induce apoptotic changes in NHDF cells following two hours of incubation (Figure 4C).

### Annexin V Staining of Apopotic Melanoma A375 Cells

**Figure 6:**
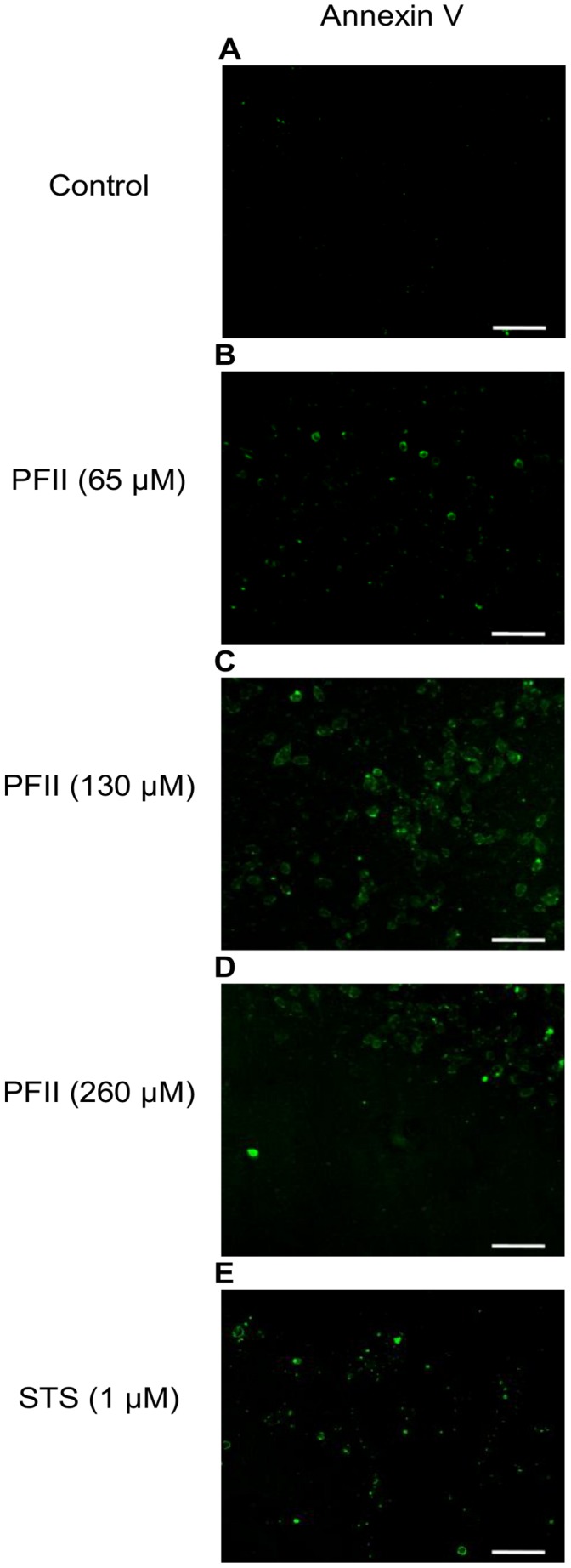
Detection of phosphatydylserine on the outer cellular membrane with annexin V. A375 cell were incubated with different concentrations of PFII for 24 h and non-fixed living cells were labeled with annexin V-Alexa Fluor 488. The labeled cells were immediately analyzed under a laser scanning confocal microscope (LSCM). Scale bar -100 µm.

Labeling with fluorescent annexin V was used to detect the presence of phosphatidylserine in the external layer of the plasma membrane. After treatment with PSII at concentrations (65, 130 and 260 µM) for 24 h a large number melanoma A375 cells were positively stained by annexin V-fluorescein (Figure 6B–D), whereas we did not observe this effect in NHDF cells (Figure 4E). Staurosporine at a concentration of 1 µM caused annexin V labeling in a manner similar to that observed with PFII (Figure 6E).

### Membrane Integrity

**Figure 7:**
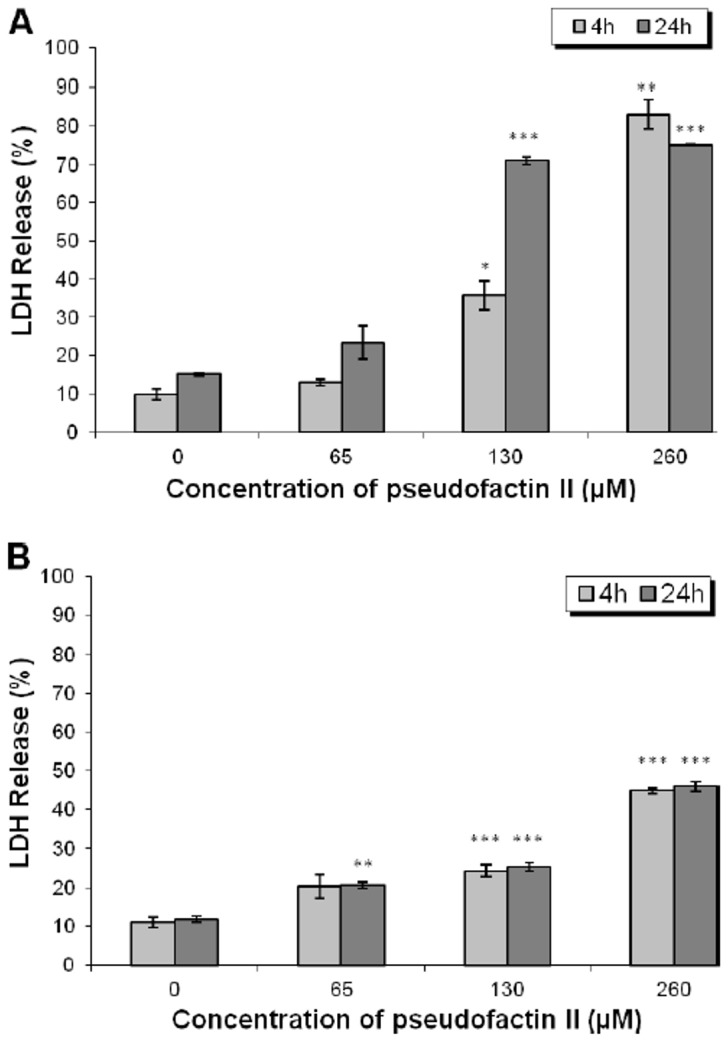
Effect of PFII on induced lactate dehydrogenase (LDH) release in A375 and NHDF cells. Melanoma A375 cells (A) and NHDF cells (B) were treated with 65, 130, 260 µM PFII for 4 and 24 h and LDH release was measured. The results are presented as the means ± SD of three independent experiments. *0.05>*P*>0.01, **0.01>*P*>0.001, ***P<0.001.

Cell death may be associated with impairment of membrane integrity resulting from interaction with biosurfactants (monomeric or micelles). This was examined by monitored the release of lactate dehydrogenase (LDH) into the extracellular milieu following exposure to increasing concentrations of PFII for 4 or 24 h (Figure 7). PFII supplementation above a concentration 130 µM increased cell death in melanoma cells. The cytotoxic effect of PFII at concentration of 130 µM intensified with exposure time for melanoma cells, but not for NHDF cells. At a PFII concentration of 260 µM nearly all melanoma cell were killed within 4 h, while 50% of NHDF cells were still viable after 24 h.

### CMC Evaluation in Culture Medium

To explain the increase of pro-apoptotic activity at concentrations above 130 µM, PFII surface tension was measured in DMEM and α-MEM medium by the du Nouy’s ring method [19]. The critical micelle concentration (CMC) was estimated to be in the range 130–140 µM. This suggests that the mechanisms of apoptosis induction by PFII in melanoma cells may be strongly dependent on micelles formation.

### Measurements of Mean Diameter of PFII Micelles

In this study biosurfactant-PFII was applied at concentrations higher than the critical micellar concentrations so as to relate their respective micellar properties to their potential proapoptotic effects. The physicochemical properties of the polymeric micelles were examined in terms of particle size and polydispersity index (PDI) (data not shown). The mean hydrodynamic diameter of PFII micelles in water was ranged from 40.2 nm to 60.3 nm, while PDI values ranged from 0.137 to 1.0.

## Discussion

The lipopeptide biosurfactant pseudofactin II induces apoptosis of melanoma A375 cells, while normal human dermal fibroblast are much less affected under the same experimental conditions. The mechanism of apoptosis induction may be based on the membrane permeabilisation [21] resulting from the interaction of PFII micelles with the plasma membrane, as evidenced by the release of LDH into the culture medium (Figure 7). Thus, the mode of action of PFII is different from pro-apoptotic activity of the previously described cyclic lipopeptide (CLP) [15] which did not affected membrane integrity.

Impairment of membrane integrity may lead to the observed Ca^2+^ influx, which triggers further signaling cascades, like measured caspase-3 activation, that finally lead to apoptosis. This hypothesis of the mechanism of melanoma cells death by PFII is further supported by the non-linear increase in cytotoxic activity of PFII above CMC. Usually the CMC of surfactants is measured in water, as it was previously determined for PFII [19]. However, due to interactions PFII with cell culture medium components, CMC measured in our experimental conditions was about 2-fold higher (130–140 µM) than previously measured in water. This concentration correlated very well with the increase of PFII pro-apoptotic activity (Figure 1). While the suggested mechanism of action seems very likely, PFII specificity against melanoma cells remains to be elucidated. *In vitro* studies using trehalose lipid biosurfactant suggests that it acts as a weak detergent which may prefer membrane incorporation over micellization. Trehalose lipid biosurfactant also exerts hemolytic activity [22], while this was not the case for PFII (results not shown). The specific activity of PFII toward melanoma cells could result from a variation in plasma membrane composition, for example, lower sterol content. The increasing melanoma incidence among the general population (associated with global decreases in stratospheric ozone and increased recreational exposure) is alarming [23] and is most often due to late diagnosis. The prognosis for individuals with this disease for curing is relatively poor. Thus, PFII could be considered as important role in promoting apoptosis at the very early stages of the melanoma formation or in prevention of its expansion. Thus, the PFII could be further analyzed on human skin for the ability to induce irritant or allergic contact dermatitis.

## Materials and Methods

### Cell Culture Conditions

Human A375 melanoma (ATCC CRL-1619) cells were cultured in Dulbecco’s modified Eagle’s medium (DMEM, Institute of Immunology and Experimental Therapy (IITD), Poland), adjusted to contain 1.5 g/l sodium bicarbonate and 4.5 g/l glucose supplemented with 5% foetal bovine serum (FBS) and antibiotics (10 U/ml penicillin and 10 µg/ml streptomycin). Normal Human Dermal Fibroblasts (NHDF) (Lonza, Basel, Switzerland) and cultured in α-Minimum Essential Medium (α-MEM, IITD, Poland) supplemented with 10% FBS and antibiotics. Normal Human Epidermal Keratinocytes (NHEK) from PromoCell GmbH and grown in Keratinocyte growth medium (KGM-Gold™ from Lonza, Basel, Switzerland). Cells were maintained in humidified incubator at 37°C and 5% CO_2_. Cells were routinely passaged following trypsinization (0.25% trypsin/0.05% EDTA). For all experiments, A375 cells and NHDF cells were used at 80% of confluence following 2–6 passages.

### Cell Treatment

Melanoma A375 cells, NHDF cells and NHEK cells were treated for 6 (data not shown), 24 and 48 h with increasing concentrations of PFII (0–260 µM) added to the cell culture medium. Staurosporine (STS; Sigma, St Louis, MO, USA) added to the culture medium at concentrations of 0.01–1 µM, was used as an apoptosis positive control. PFII and STS were dissolved in DMSO to obtain concentration of 44 mg/ml and 5 mg/ml, respectively. Control observations were made to examine the influence of DMSO on A375 cell and NHDF cell morphology and/or actin cytoskeletal organization.

### Cell Proliferation (MTT Assay)

A375, NHDF and NHEK cells were seeded onto 96-well plates at a density of 1×10^4^, 3×10^3^ and 1×10^4^ cells per well, respectively. The cells were incubated for 24 and 48 h with increasing concentrations of PFII (0–260 µM). The cell proliferation rate was determined with the standard MTT (3-(4,5-dimethylthiazol-2-yl)-2,5-diphenyltetrazolium bromide) (Sigma, St Louis, MO, USA) assay procedure [24]. Measurements were made in three independent experiments, each performed in triplicate.

### Visualization of Actin Microfilaments, Caspase-3 Activity, and Nuclear Morphology

Melanoma A375 and NHDF cells grown on sterile glass coverslips were fixed with 4% paraformaldehyde (PFA) in PBS at 4°C for 20 min, rinsed (3X) with PBS, and then permeabilized with a 0.1% Triton X-100 in PBS for 20 min at room temperature (RT). The cells were rinsed with PBS and incubated in 1% bovine serum albumin in PBS for 1 h at RT.

Actin microfilaments were visualized by staining for 1 h with rhodamine-conjugated phalloidin (Sigma, St Louis, MO, USA) at a concentration of 10^–6^ M in PBS.

Active caspase-3 was labelled with monoclonal anti-caspase-3 antibody (clone 269518; R&D Systems) at a concentration of 1 µg/ml, followed by treatment with FITC (fluorescein isothiocyanate)-conjugated anti-mouse secondary antibody (Jackson ImmunoResearch) diluted in PBS (1∶200).

Apoptotic nuclear morphology of fixed cells was assessed using Hoechst 33342 (Molecular Probes, USA), which was applied at a concentration of 5 µg/ml for 10 min incubation.

The cells (coverslips) were washed with PBS, mounted with fluorescence stabilizing medium (Dako, Glostrup, Denmark). Depending on the above experiment, observations were made with an Olympus IX70 fluorescence microscope or an Olympus FV 500 laser scanning confocal microscope (LSCM).

### DNA Fragmentation

A375 melanoma cells (3×10^5^ cells) were seeded in 6-well microtiter plates and cultured in DMEM medium supplemented with vehicle (DMSO 1%) or DMSO containing PFII (65, 130, 260 µM) for 24 h at 37°C. Staurosporine (STS), a classic inducer of apoptosis, was used as a positive control. Cells were treated in medium containing 1 µM STS for 2 h at 37°C. After treatment with PFII or STS, the cells were scraped from the dishes using a rubber policeman and centrifuged at 9000×g for 5 min at 4°C. The pellet was washed (2X) with ice-cold PBS and the DNA extracted using a NucleoSpin Tissue kit (Macherey-Nagel, Düren, Germany) according to the manufacturer’s instructions. The DNA fragments obtained were electrophoretically separated using a 1.5% agarose gel. Gels were stained with ethidium bromide and photographed under UV transillumination.

### Laser Scanning Confocal Microscope Analysis of [Ca^2+^]i

A laser scanning confocal microscope was used for analysis of [Ca^2+^]i in A375 and NHDF cells. The cells were grown on coverslips (or 96 well plates) and treated with 65, 130, 260 µM of PFII for 2, 8 and 24 h. Following incubation Fluo-3/AM (Sigma, St Louis, MO, USA) was added to the culture medium to the final concentration of 1 µM, the cells incubated for 30 min at 37°C, and then washed with PBS. To determine the [Ca^2+^]i levels, LSCM analysis was performed with an Olympus FV 500 imaging system with an argon laser (**λ**_EX_ 506 nm, **λ**_EM_ 526 nm). As a positive control (data not shown), untreated cells were incubated with 5 µM Calcium Ionophore A-23187 (Sigma, St Louis, MO, USA) and immediately analysed under LSCM.

### Detection of Apoptosis with the Annexin V-Alexa Fluor 488 Assay

Cells grown on glass coverslips were treated with PFII (65, 130, 260 µM) for 24 h, washed in cold phosphate buffered saline (PBS), and then washed in annexin-binding buffer (10 mM HEPES, 140 mM NaCl, and 2.5 mM CaCl_2_, pH 7.4). The coverslips with attached cells were placed in 50 µL of Annexin V conjugated with Alexa Fluor 488 (Molecular Probes, USA) in annexin-binding buffer according manufacturer’s protocol (1∶20) for 15 minutes at RT, washed with annexin-binding buffer, and immediately analysed with LSCM, using an argon laser (λ_EX_ 496 nm, λ_EM_ 524).

### Lactate Dehydrogenase (LDH) Assay

The release of LDH from damaged cells was measured with CytoTox-ONE (Promega, MA, USA) homogeneous membrane integrity assay. Melanoma A375 and NHDF cells were seeded onto 96-well plates at a density of 1×10^4^ and 3×10^3^ cells per well, respectively. Cells were maintained in humidified incubator at 37°C and 5% CO_2_. The cells were incubated for 2 and 24 h with increasing concentrations (65, 130, 260 µM) of PFII added to the cell culture medium. Maximum LDH release was determined by adding 2 µl of the CytoTox-ONE lysis solution to control wells for 10 min. The assay was performed in 96-well plates by adding 100 µl of the sample supernatant and 100 µl of CytoTox-ONE reagent, after which the plate was shaken for 10 s. After 10 min of incubation, 50 µl CytoTox-ONE stop solution was added and the plate was again shaken for 10 s. The fluorescent signal was measured with a Cary Eclipse Spectrofluorimeter (Varian, Cary, NC) with λ_EX_ 560 nm, λ_EM_ 590. LDH-release was calculated as percentage of LDH released in the culture media of total LDH (media and lysates).

### Physico-chemical Properties

Surface tension was determined with a Kruss K100 Tensiometer (Kruss GmbH, Hamburg, Germany) at 37°C, according to the du Nouy’s ring method [25].

### Measurement of Micelle Size

The mean particle size and polydispersity index (PDI) of PFII diluted in Milli-Q water were determined using a Zetasizer Nano-ZS (Malvern Instruments Ltd., Malvern, UK) and PCS software. The analysis of particle size and PDI, determined by photon correlation spectroscopy, was performed using the volume distribution algorithm. The polydispersity index qualifies the particle size distribution, which here ranged from 0 for monodispersed to 1.0 for entirely heterodispersed emulsions. In order to obtain the optimum light scattering intensity for the size analysis, approximately 100 µl of the PFII was added to 900 µl of Milli-Q water. All the measurements were carried out at 25°C.

### Statistical Analysis

All data are presented as mean ± standard deviation (SD). Statistical significance was determined using Student’s *t* test. The significance level was set at *P*<0.05.
